# A Novel Model of Intravital Platelet Imaging Using CD41-ZsGreen1 Transgenic Rats

**DOI:** 10.1371/journal.pone.0154661

**Published:** 2016-04-29

**Authors:** Makoto Mizuno, Atsuyuki Tomizawa, Kousaku Ohno, Joseph A. Jakubowski, Atsuhiro Sugidachi

**Affiliations:** 1 Biological Research Laboratories, Daiichi Sankyo Co., Ltd., Tokyo, Japan; 2 Lilly Research Laboratories, Eli Lilly and Company, Indianapolis, IN, United States of America; National Cerebral and Cardiovascular Center, JAPAN

## Abstract

Platelets play pivotal roles in both hemostasis and thrombosis. Although models of intravital platelet imaging are available for thrombosis studies in mice, few are available for rat studies. The present effort aimed to generate fluorescent platelets in rats and assess their dynamics in a rat model of arterial injury. We generated CD41-ZsGreen1 transgenic rats, in which green fluorescence protein ZsGreen1 was expressed specifically in megakaryocytes and thus platelets. The transgenic rats exhibited normal hematological and biochemical values with the exception of body weight and erythroid parameters, which were slightly lower than those of wild-type rats. Platelet aggregation, induced by 20 μM ADP and 10 μg/ml collagen, and blood clotting times were not significantly different between transgenic and wild-type rats. Saphenous arteries of transgenic rats were injured with 10% FeCl_3_, and the formation of fluorescent thrombi was evaluated using confocal microscopy. FeCl_3_ caused time-dependent increases in the mean fluorescence intensity of injured arteries of vehicle-treated rats. Prasugrel (3 mg/kg, p.o.), administered 2 h before FeCl_3_, significantly inhibited fluorescence compared with vehicle-treated rats (4.5 ± 0.4 vs. 14.9 ± 2.4 arbitrary fluorescence units at 30 min, respectively, n = 8, *P* = 0.0037). These data indicate that CD41-ZsGreen1 transgenic rats represent a useful model for intravital imaging of platelet-mediated thrombus formation and the evaluation of antithrombotic agents.

## Introduction

Formation of a thrombus in major arteries occurs in association with arteriosclerosis and is one of the leading causes of morbidity and mortality in industrialized countries [1]. Although various factors involved in life-threatening atherothrombotic processes are known and are targeted successfully with drugs [2, 3], unknown mechanisms must contribute to the pathogenesis of atherothrombosis. Atherothrombosis plays a critical role in many diseases, and understanding of the mechanisms involved in thrombus formation and progression may help in the prevention or treatment of myocardial infarction and stroke.

Among characterized atherothrombotic processes, platelets play several important roles [4–9]. Since platelet aggregation was first measured using a turbidimetric method by Born [10], considerable progress has been made in understanding platelet aggregation and its control. However, the precise pathogenesis of thrombus formation in blood vessels requires further elucidation since it is difficult to extrapolate from *in vitro* findings to *in vivo* animal models and human disease states. Thus, for investigating platelet involvement *in vivo* physiological studies are advantageous [11]. Real-time visualization of thrombus formation provides spatiotemporal information about events within the growing thrombus in the living animal. In rodent models, platelet thrombi are visualized using probes such as fluorescence-conjugated platelet antibodies or platelets labeled with fluorescent dyes [12–15]. These intravital fluorescence imaging techniques were mainly adapted for mouse studies; however, there are few suitable *in vivo* models for imaging platelets of rats. Species differences, such as receptor distribution and drug reactivity, between rats and mice have been reported in several reports [16, 17]. Platelet reactivity of rats also appears to be different from that of mice. Further, Nylander et al. (2006) have reported that certain aspects of human platelet reactivity are more similar to those of rats than mice [18]. In addition to these species differences, since the body size of rats is larger than that of mice, several more elaborate thrombotic disease models are more easily created in rats compared to mice. Therefore, rat models of intravital imaging to evaluate *in vivo* platelet function will be of value.

The integrin CD41/CD61 (GPIIb/IIIa) plays a central role in platelet aggregation [19], and specific therapeutic antibodies are used clinically [20]. GPIIb/IIIa is a marker for hematopoietic differentiation and is specifically expressed by the mature megakaryocytic lineage, and thus in platelets [21]. Moreover, the promoter of the gene encoding CD41 is widely used to express megakaryocyte- and platelet-specific targeted proteins [22–24]. For example, Zhang et al. [22] generated CD41-yellow fluorescent protein (YFP) transgenic mice that express YFP under the control of the mouse promoter of the gene encoding the GPIIb-subunit of GPIIb/IIIa. In the present study, we adopted a similar strategy using the CD41 promoter of rats to specifically express a new fluorescent protein, ZsGreen1 [25–27], in platelets/megakaryocytes.

Thus the purpose of the present study was to generate fluorescent rat platelets and assess their dynamics in arteries injured by FeCl_3_. Further, we assessed the effect of the antiplatelet agent prasugrel on rat platelet dynamics and thrombus imaging at sites of arterial injury using intravital platelet thrombus imaging.

## Results

### Generation of CD41-ZsGreen1 transgenic rats

**Fig 1:**
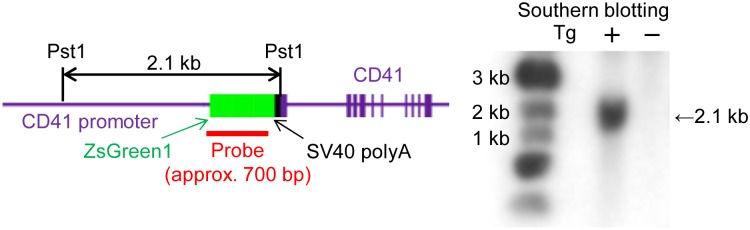
Analysis of the transgene structure in the genome of CD41-ZsGreen1 transgenic rats. (A) Schematic of transgene constructs and hybridization probe for Southern blot analysis. The start codon of *Cd41* was replaced by the ZsGreen1 fragment. (B) Southern blot analysis of tail DNA samples of CD41-ZsGreen1 transgenic rats. Genomic DNA isolated from the tail was digested with PstI, electrophoresed through an agarose gel, and transferred to a nylon membrane. The nylon membrane was hybridized to the ZsGreen1 probe to detect the 2.1-kb restriction fragment.

Several lines of CD41-ZsGreen1 transgenic rats were generated and analyzed by Southern blotting to confirm the presence of the transgene in genomic DNA. When CD41-ZsGreen1 transgenic rat DNA was digested with PstI and hybridized to the ZsGreen1 probe described above, a 2.1 kb band was detected (Fig 1). We analyzed the genomic DNAs of 19 transgenic founder lines using Southern blot analysis and performed flow cytometric analysis of platelets to select 10 lines for breeding. One founder line was selected because of its high percentage of ZsGreen1-positive platelets and its lack of detectable abnormal hematological, biochemical, or platelet aggregation values.

### Flow cytometry

**Fig 2:**
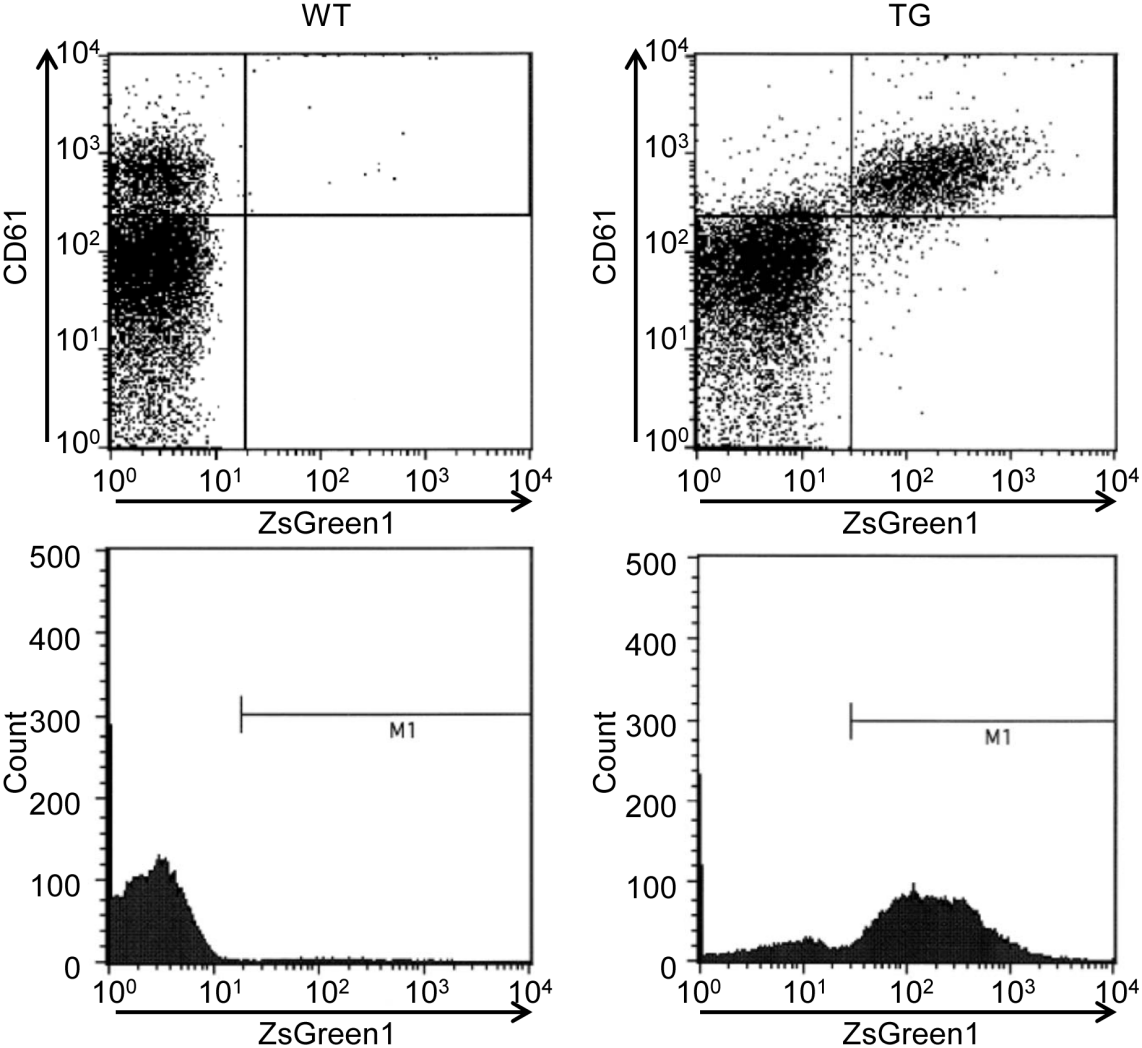
Representative flow cytometric analysis of CD41-ZsGreen1 transgenic rats. EDTA-treated blood samples were withdrawn from the abdominal vein for flow cytometric analysis. The blood was mixed with phycoerythrin-labeled hamster anti-mouse CD61 antibody. Flow cytometric analyses were performed using a FACSCalibur. M1: ZsGreen1-positive platelets. WT: wild-type Sprague-Dawley rats, TG: CD41-ZsGreen1 transgenic rats.

To assess ZsGreen1 expression in platelets, flow cytometric analysis was performed in wild-type and transgenic rats (Fig 2). Sprague-Dawley (SD) rats were used as wild-type rats. In wild-type rats, a low level of ZsGreen1 fluorescence was detected. However, transgenic rats emitted more intense ZsGreen1 fluorescence. The percentage of CD61-positive platelets that expressed ZsGreen1 was 83.75% ± 0.59%. ZsGreen1 expression was not detectable at levels above baseline in other blood cells.

### Characteristics of CD41-ZsGreen1 transgenic rats

We determined the general condition, hematological parameters, and biochemical parameters of CD41-ZsGreen1 transgenic rats that were 8 weeks of age. The general condition of CD41-ZsGreen1 transgenic rats did not differ significantly from that of wild-type rats except for body weight (236.0 ± 2.6 g vs. 272.7 ± 2.6 g). Hematological data are shown in Table 1. The values of erythrocyte count, hematocrit, and hemoglobin concentration of CD41-ZsGreen1 transgenic rats were significantly lower and reticulocyte count was significantly higher than those of wild-type rats (erythrocyte count: 6.613 ± 0.093 × 10^6^/μl vs. 7.710 ± 0.127 × 10^6^/μl; hemoglobin concentration: 12.8 ± 0.2 g/dl vs. 14.5 ± 0.2 g/dl; hematocrit: 38.57% ± 0.54% vs. 43.43% ± 0.62%; reticulocyte: 6.467% ± 0.067% vs. 3.133% ± 0.120%, respectively). The values of other hematological measures were similar between wild-type and CD41-ZsGreen1 transgenic rats. Since, in the initial experiments a small number of animals were studied, we further evaluated these parameters in a subsequent study of wild-type and CD41-ZsGreen1 transgenic rats. The findings were as follows: body weight was 248.3 ± 2.4 g for wild-type rats and 198.8 ± 11.6 g for CD41-ZsGreen1 transgenic rats (n = 6 for each, *P* = 0.0019); erythrocyte counts were 7.93 ± 0.20 × 10^6^/μl for wild-type rats (n = 6) and 7.54 ± 0.13 × 10^6^/μl for CD41-ZsGreen1 transgenic rats (n = 5, *P* = 0.1467), confirming that CD41-ZsGreen1 transgenic rats exhibited a small, but significantly difference in body weight and a tendency to lower erythrocyte count.

**Table 1:** Hematological parameters of CD41-ZsGreen1 transgenic rats.

	WT	TG
Erythrocytes (10^6^/μl)	7.71 ± 0.13	6.61 ± 0.09*
Hemoglobin (g/dl)	14.5 ± 0.2	12.8 ± 0.2*
Hematocrit (%)	43.4 ± 0.6	38.6 ± 0.5*
Mean corpuscular volume (fl)	56.4 ± 0.8	58.3 ± 1.3
Reticulocytes (%)	3.1 ± 0.1	6.5 ± 0.1*
Platelets (10^3^/μl)	807 ± 19	898 ± 54
Leukocytes (10^3^/μl)	5.38 ± 0.20	6.18 ± 0.47
% Platelet aggregation (ADP 20 μM)	64 ± 2	68 ± 1
% Platelet aggregation (collagen 10 μg/ml)	69 ± 1	73 ± 1
Prothrombin time (s)	10.5 ± 0.0	10.5 ± 0.1
Activated partial thromboplastin time (s)	14.9 ± 0.5	15.2 ± 0.5

Blood samples were withdrawn from the abdominal vein under anesthesia. Hematological parameters were analyzed using a hematology analyzer. Blood clotting times were analyzed using an automated coagulation analyzer. Platelet aggregation induced by ADP (20 μM) and collagen (10 μg/mL) was measured using light transmission aggregometry. Data are expressed as the mean ± standard error of the mean (SEM) of 3 rats/group.

*P < 0.05 compared with WT rats (Student’s *t* test).

WT: wild-type Sprague-Dawley rats, TG: CD41-ZsGreen1 transgenic rats, ADP: adenosine 5’-diphosphate.

Biochemical data are shown in Table 2. CD41-ZsGreen1 transgenic rats exhibited normal biochemical values with the exception of plasma glucose levels, which were significantly lower compared with those of wild-type rats (179.7 ± 2.3 mg/dl and 221.7 ± 6.1 mg/dl, respectively). There were small changes in the values of some parameters described above that were within the normal range, indicating that the phenotypes of CD41-ZsGreen1 transgenic rats were not physiologically abnormal.

**Table 2:** Biochemical parameters of CD41-ZsGreen1 transgenic rats.

	WT	TG
Aspartate transaminase (IU/l)	71 ± 17	64 ± 3
Alanine transaminase (IU/l)	31 ± 6	33 ± 3
Alkaline phosphatase (IU/l)	1312 ± 231	1349 ± 168
Lactate dehydrogenase (IU/l)	462 ± 20	337 ± 66
Creatine kinase (IU/l)	311 ± 39	274 ± 48
Total bilirubin (mg/dl)	0.01 ± 0.00	0.02 ± 0.00
Total protein (g/dl)	5.1 ± 0.1	4.9 ± 0.1
Phospholipid (mg/dl)	118 ± 4	141 ± 13
Triglyceride (mg/dl)	37 ± 9	33 ± 5
Total cholesterol (mg/dl)	74 ± 6	87 ± 9
Glucose (mg/dl)	222 ± 6	180 ± 2*
Urea nitrogen (mg/dl)	12.4 ± 1.2	12.2 ± 0.3
Creatinine (mg/dl)	0.26 ± 0.01	0.27 ± 0.01
Inorganic phosphorus (mg/dl)	7.85 ± 0.57	8.74 ± 0.87
Ca (mg/dl)	9.9 ± 0.1	10.1 ± 0.0
Na (mEq/l)	142 ± 1	142 ± 1
K (mEq/l)	4.5 ± 0.1	4.4 ± 0.1
Cl (mEq/l)	105 ± 1	104 ± 1
Albumin (g/dl)	2.8 ± 0.1	2.8 ± 0.0
Globulin (g/dl)	2.3 ± 0.0	2.1 ± 0.1
Albumin/Globulin	1.27 ± 0.03	1.34 ± 0.03

Blood samples were withdrawn from the abdominal aorta under anesthesia. Blood biochemistry was analyzed using an automated biochemical analyzer. Data are expressed as the mean ± standard error of the mean (SEM) of 3 rats/group.

*P < 0.05 compared with WT rats (Student’s *t* test).

WT: wild type Sprague-Dawley rats, TG: CD41-ZsGreen1 transgenic rats.

### Histological analysis

**Fig 3:**
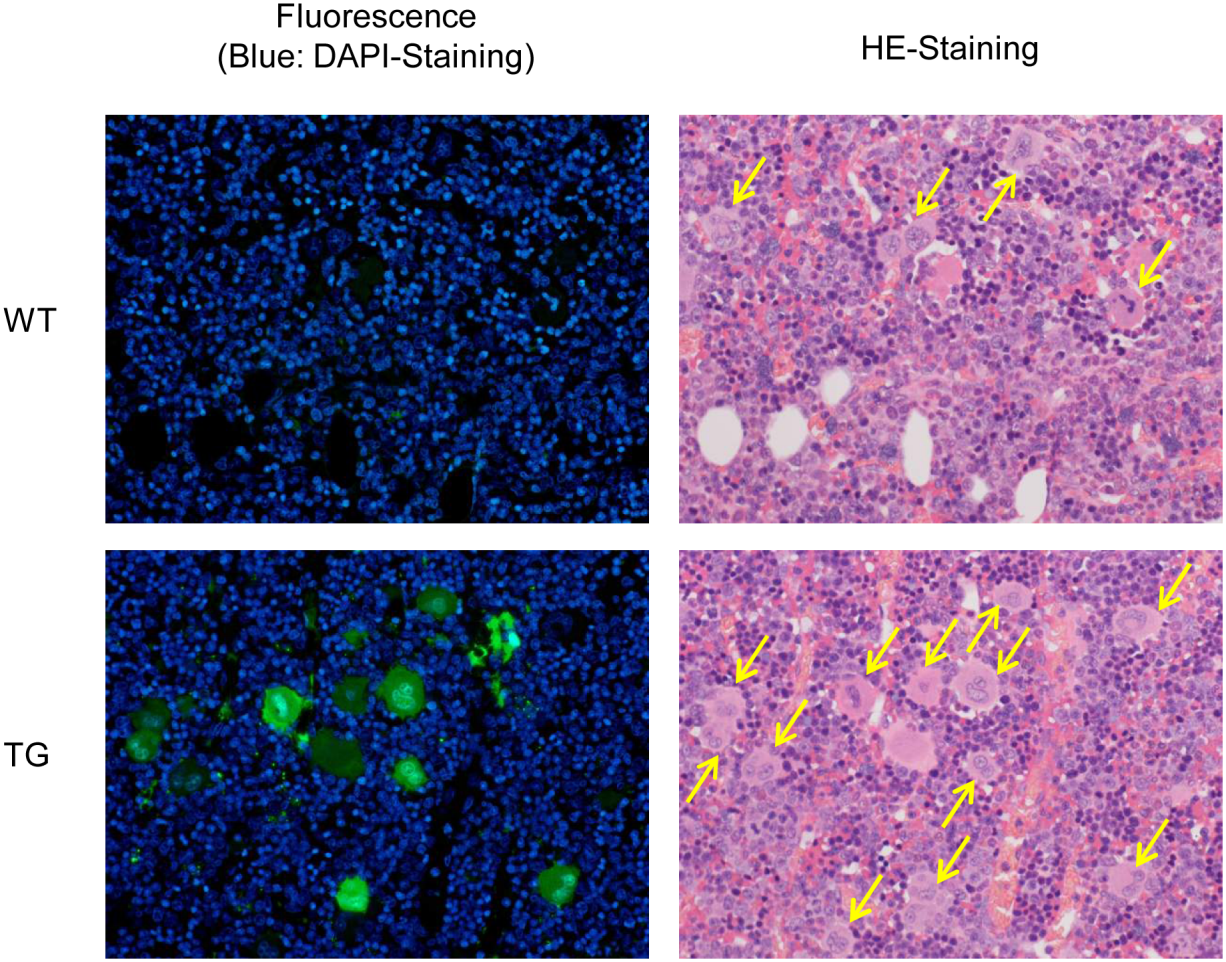
Specific expression of green fluorescent protein in bone marrow megakaryocytes of CD41-ZsGreen1 transgenic rats. Bone marrow from the femur was excised, fixed with 10% buffered formalin, and embedded in paraffin. Sections were prepared and stained with DAPI or HE. Arrows in the HE photograph indicate megakaryocytes. WT: wild-type Sprague-Dawley rats, TG: CD41-ZsGreen1 transgenic rats, HE: hematoxylin-eosin, DAPI: 4′,6-diamidino-2-phenylindole.

ZsGreen1 expression was analyzed by measuring the fluorescence of the bone marrow, spleen, liver, and thymus of CD41-ZsGreen1 transgenic rats. ZsGreen1 was expressed in the bone marrow only by megakaryocytes (Fig 3) and by Kupffer cells in the liver. These findings are consistent with phagocytosis of aging platelets by Kupffer cells. Fluorescence above the background level was not detected in other cell types.

### Platelet aggregation and blood clotting times

**Fig 4:**
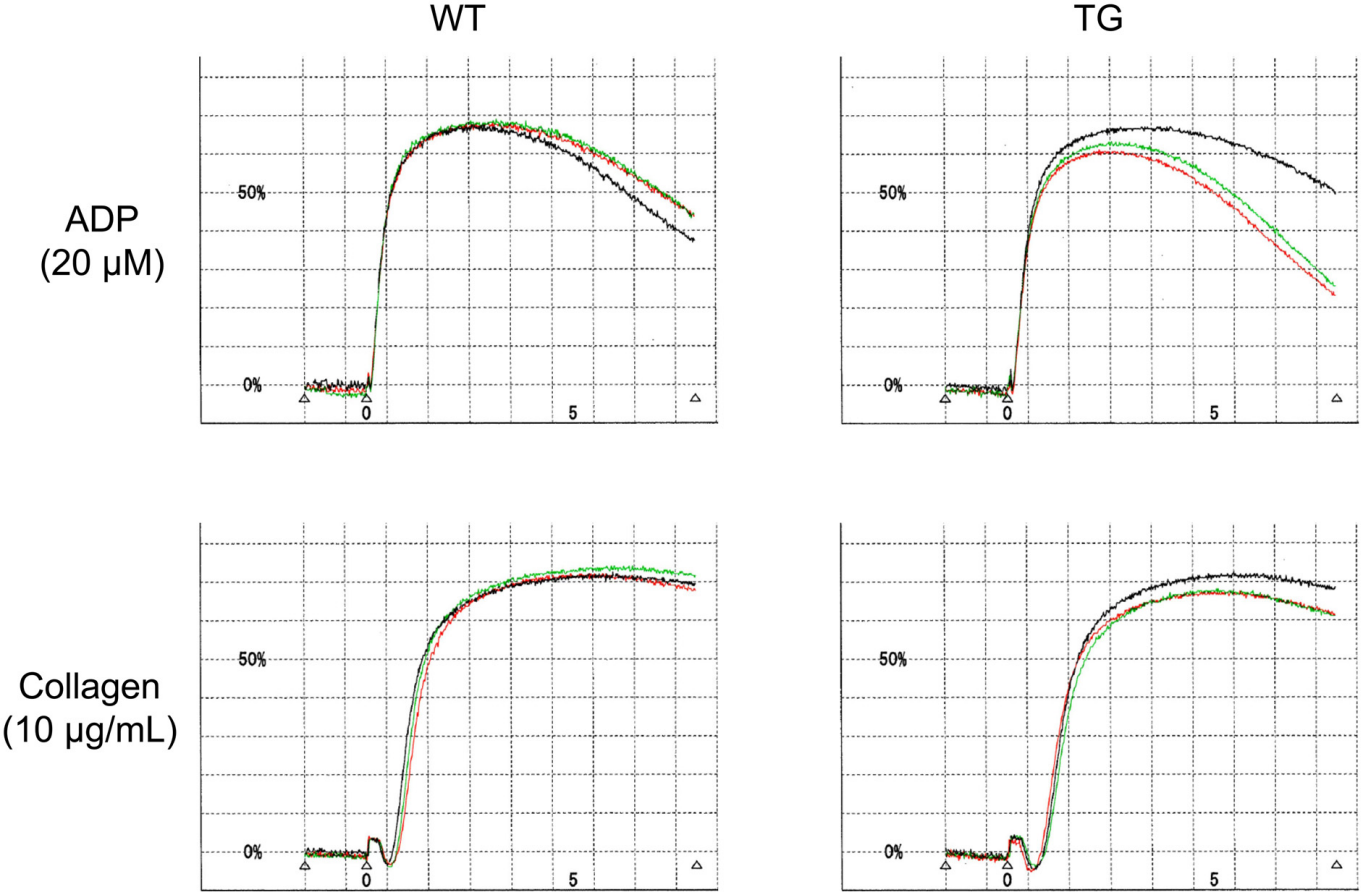
Representative ADP- and collagen-induced platelet aggregation tracings of CD41-ZsGreen1 transgenic rats. Citrated blood was collected from the abdominal aorta and centrifuged to obtain platelet-rich plasma. Platelet aggregation induced by ADP (20 μM) and collagen (10 μg/mL) was measured using light transmission aggregometry. WT: wild-type Sprague-Dawley rats, TG: CD41-ZsGreen1 transgenic rats, ADP: adenosine 5’-diphosphate.

Platelet aggregation induced by ADP and collagen as well as blood clotting times are shown in Table 1. Platelet aggregation tracings are shown in Fig 4. Platelet aggregation induced by ADP and collagen and blood clotting times (prothrombin time and activated partial thromboplastin time) were not significantly different between CD41-ZsGreen1 transgenic and wild-type SD rats.

### Intravital imaging

**Fig 5:**
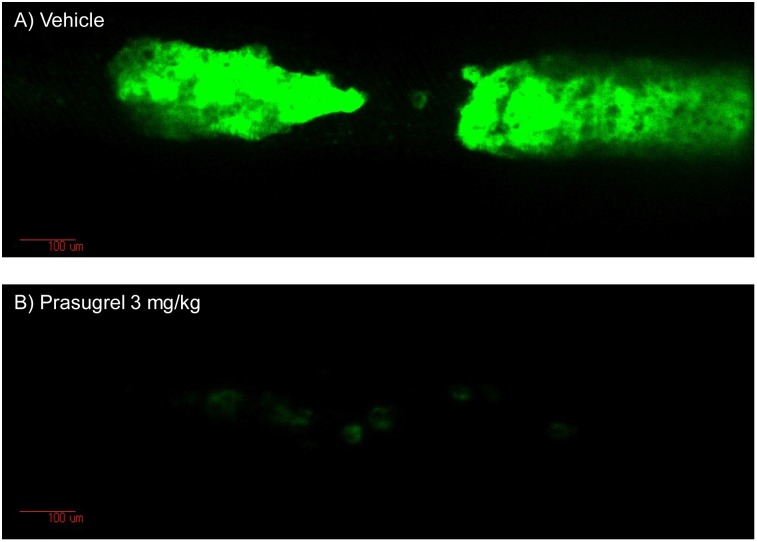
Representative images of platelet-rich thrombus induced by 10% FeCl_3_ in the saphenous artery of CD41-ZsGreen1 transgenic rats 30 min after FeCl_3_ application. Male CD41-ZsGreen1 transgenic rats were anesthetized, the left saphenous artery was exposed, and a filter paper, presoaked with 10% FeCl_3_ was attached for 3 min to induce thrombosis 2 h after administration of vehicle or prasugrel. Fluorescence was measured every 1 s for 25 min using a confocal laser microscope.

In a preliminary study of CD41-ZsGreen1 transgenic rats, individual platelets were detected in small vessels such as the mesenteric and cremaster muscle vessels. Further, individual circulating platelets were detected in large vessels, including the saphenous vessels and carotid artery (S1 Video). We confirmed thrombus formation in several thrombosis models, such as laser-induced, and photochemically-induced thrombosis in the saphenous artery and vein, mesenteric arterioles, and cremaster arterioles of CD41-ZsGreen1 transgenic rats. Typical static vascular images (30 min after FeCl_3_ treatment) are shown in Fig 5. Typical video images of thrombus formation induced by FeCl_3_ in vehicle- and prasugrel-treated rats are shown in S2 and S3 Videos, respectively.

**Fig 6:**
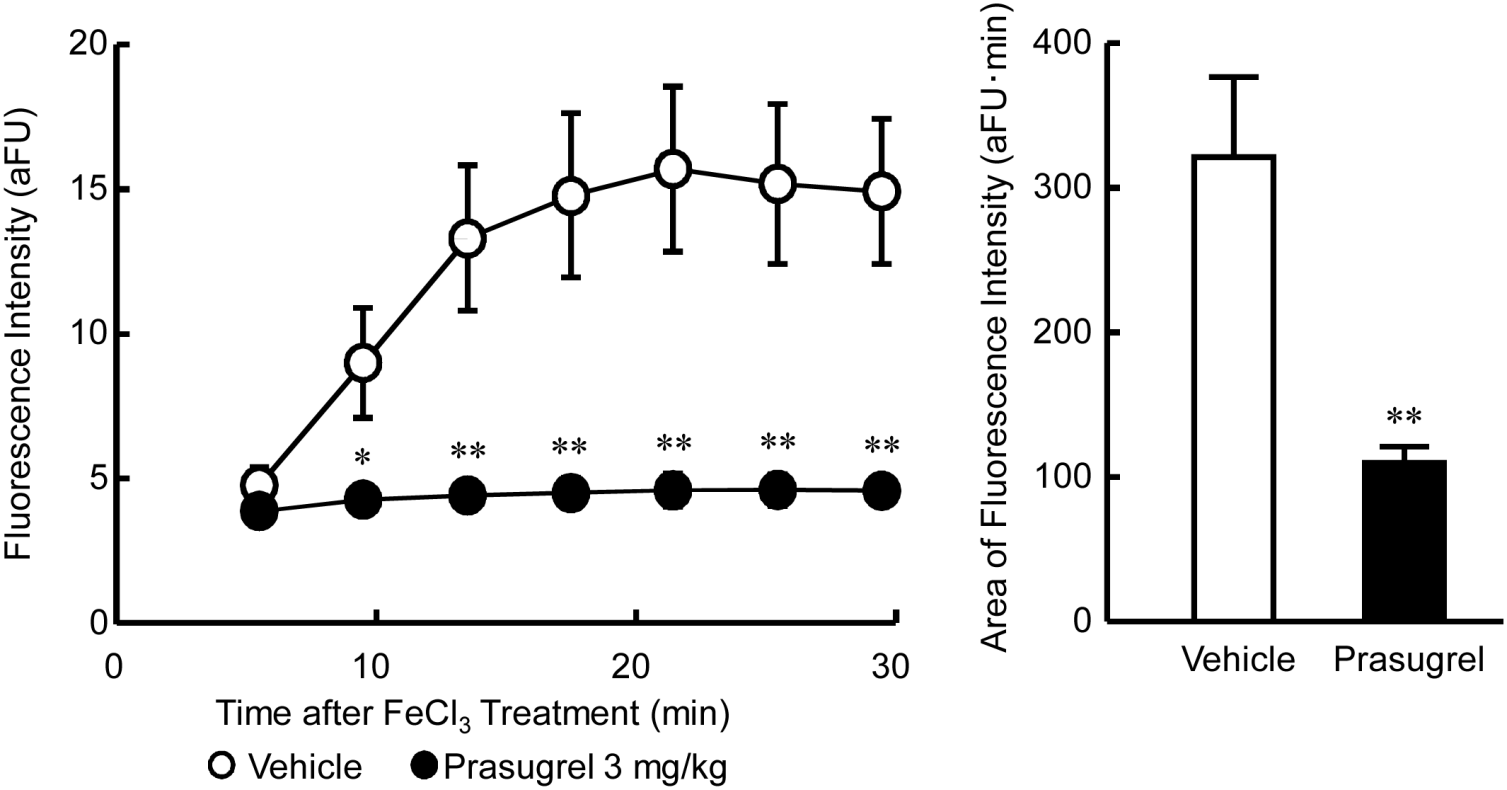
Fluorescence accumulation induced by 10% FeCl_3_ applied to the saphenous arteries of CD41-ZsGreen1 transgenic rats. Male CD41-ZsGreen1 transgenic rats were anesthetized, the left saphenous artery was exposed, and a filter paper, presoaked with 10% FeCl_3_ was attached for 3 min to induce thrombosis 2 h after administration of vehicle or prasugrel. Fluorescence was measured every 1 s for 25 min using a confocal laser microscope. (A) Time course of fluorescence intensity. (B) Area of fluorescence accumulation. Data are expressed as the mean ± standard error of the mean (SEM) of 8 rats/group. *P < 0.05 compared with vehicle-treated rats (Welch’s *t* test). aFU: arbitrary fluorescence unit.

Treatment of the saphenous artery with 10% FeCl_3_ in vehicle animals increased fluorescence intensity only in the FeCl_3_-treated area after approximately 10 min of reflecting platelet-rich thrombus formation in the injured vessel. Platelet thrombus formation reached its maximum within approximately 20 min after FeCl_3_ treatment. Variability of the averaged fluorescence intensity was relatively high due to differences among the times required to form the thrombi. However, the platelet thrombi grew rapidly once they began to grow in each vehicle-treated rat. In some rats, platelet thrombi repeatedly detached and re-formed. Fluorescence intensities 5 min after FeCl_3_ treatment were not significantly different between the vehicle and prasugrel groups (4.72 ± 0.59 arbitrary fluorescence unit [aFU]∙min, 3.83 ± 0.07 aFU∙min, respectively, *P* = 0.1762), suggesting non-P2Y_12_ pathways might be involved in the initiation of platelet aggregation. However, prasugrel significantly inhibited the formation of platelet thrombi starting 9 min after FeCl_3_ application (Fig 6A). The fluorescence intensities 30 min after FeCl_3_ application in the vehicle- and prasugrel-treated rats were 14.9 ± 2.4 aFU and 4.5 ± 0.4 aFU (*P* = 0.0037), respectively. The total areas of fluorescence of the vehicle and prasugrel groups were 321.70 ± 52.80 aFU∙min and 109.85 ± 10.20 aFU∙min (*P* = 0.0049), respectively (Fig 6B).

## Discussion

In the present study, we generated CD41-ZsGreen1 transgenic rats, which specifically expressed ZsGreen1 fluorescent protein in megakaryocytes and platelets. All physiological parameters that we assessed during the observation period were within normal laboratory ranges for wild-type rats. However, body weight of CD41-ZsGreen1 transgenic rats was slightly but significantly lower than that of wild-type rats, and erythroid parameters of CD41-ZsGreen1 transgenic rats tended to be lower than those of wild-type rats. The precise mechanism(s) that reduced body weight and reduced erythrocyte counts is unknown. Possible factors influencing these differences might be random insertion of the BAC transgene into the genome, unknown ZsGreen1 function, littermates not used as wild-type rat controls, etc. Since similar trends were observed in separate experiments and in other experiments using other lines of CD41-ZsGreen1 transgenic rats, the possibility of involvement of random BAC gene insertion in mediating lower body weights and erythrocyte parameters is considered low. Further studies are required to address this finding. Importantly, we determined that the ability of platelets to aggregate and blood clotting times, factors central to thrombus formation, were similar between wild-type and transgenic rats. Taken together, we conclude that CD41-ZsGreen1 transgenic rats may provide a valuable model for use in further intravital imaging studies of platelet function and its control.

Previous intravital platelet imaging studies have been done in mice and mainly employed antibodies conjugated to fluorescent reporters [14, 15, 28]. To our knowledge, few rat intravital platelet imaging studies have been published [29]. In the present effort, we initially attempted to label/detect platelets in rats using several commercially available fluorescent antibodies such as fluorescein-conjugated anti-CD41, anti-CD42b, and anti-integrin β3 antibodies. However, we were not successful in detecting platelet thrombi. Therefore, we generated transgenic rats, which specifically expressed the fluorescent protein ZsGreen1 [27] in megakaryocytes and thus platelets, reflecting our strategy to employ the CD41 promoter [22].

We show here, using flow cytometric and histological analyses, megakaryocyte- and platelet-specific expression of the ZsGreen1 protein. We successfully detected individual circulating platelets and platelet aggregates in large arteries such as the saphenous and carotid arteries in the CD41-ZsGreen1 transgenic rats. Since ZsGreen1 is a green fluorescent protein derived from *Zoanthus* fluorescent protein and emits intense fluorescence [27], individual circulating platelets could be detected. These data suggest that the CD41-ZsGreen1 transgenic rats with intense megakaryocyte- and platelet-specific fluorescence will be of value for intravital platelet imaging studies of rats.

The effects of platelet inhibitors on thrombus formation have been evaluated in traditional thrombosis models such as arterio-venous shunt models [30], photochemically-induced thrombosis models [31], and FeCl_3_-induced thrombosis models [32]. Among these studies, the FeCl_3_-injury model is one of the most intensively used for investigating the properties of antithrombotic agents, but is rarely used for intravital imaging studies. Therefore, in the present study, we investigated the effects of prasugrel, the third-generation thienopyridine antiplatelet agent, on the FeCl_3_-induced thrombosis in this novel model of intravital platelet thrombus imaging. Similar to our previous experience with more traditional rat models of thrombosis [33–35], 3 mg/kg of prasugrel completely inhibited thrombus formation induced by FeCl_3_, confirming the potent antithrombotic effect of prasugrel and the ability to detect this in the CD41-ZsGreen1 transgenic rats. Further, our present results indicate the ability of the model to follow the time-dependency of platelet thrombus formation and its inhibition.

In traditional models, thrombus formation is typically evaluated at one time point in each animal. The data presented here indicate that the CD41-ZsGreen1 transgenic rat model allows for the temporal determination of platelet participation in thrombus formation and for the evaluation of antithrombotic agents. In the present study, we used prasugrel as the only antithrombotic agent and used only FeCl_3_ to induce thrombus. Further studies are therefore required to evaluate the effect of other antithrombotic agents in other models of thrombus formation.

## Conclusions

In conclusion, we generated a CD41-ZsGreen1 transgenic rat model to study intravital platelet imaging and used it to demonstrate its promise for the evaluation of the mechanism of thrombosis and the effects of antithrombotic agents.

## Experiments

### Ethics statement

This study was carried out in strict accordance with the guidelines of the Institutional Animal Care and Use Committee of Daiichi Sankyo Co., Ltd. and the animal welfare bylaws of Shin Nippon Biomedical Laboratories, Ltd., Drug Safety Research Laboratories. The protocol was approved by the Institutional Animal Care and Use Committee of Daiichi Sankyo (Permit Number: A1402955) and by the Institutional Animal Care and Use Committee of Shin Nippon Biomedical Laboratories (Permit Number: IACUC315-125). All surgery was performed under isoflurane anesthesia, and all efforts were made to minimize suffering.

### Expression vector and generation of transgenic rats

The BAC clone (CH230-412I13) containing the rat *Cd41* gene from −92.7 kb to +44.9 kb was obtained from BACPAC resources center (Children’s Hospital Oakland Research Institute, Oakland, CA, USA). A DNA fragment encoded ZsGreen1 poly(A) was ligated to a DNA fragment encoded CD41, which was generated using Red/ET Recombination Technology [36]. The start codon of *Cd41* was replaced by the ZsGreen1 fragment. Fertilized eggs were collected from female SD rats (Japan SLC, Shizuoka, Japan) administered pregnant mare serum gonadotropin, and human chorionic gonadotropin to induce superovulation for pronuclear injection. The recombinant BAC clone was injected into the eggs, which were transplanted into pseudopregnant SD females to generate transgenic rats.

### Rat genotyping

The ZsGreen1 fragment was labeled with ^32^P and used as a probe for detecting the ZsGreen1 gene. Genomic DNA isolated from the tail was digested with PstI, electrophoresed through an agarose gel, depurinated with 0.25N HCl, and transferred to a nylon membrane using a capillary method in the presence of 0.4N NaOH. The nylon membrane was hybridized to the ZsGreen1 probe to detect the 2.1-kb restriction fragment. After hybridization, the membrane was washed several times and exposed to X-ray film at 4°C for 1 week.

### Flow cytometric analysis

2-({2-[bis(carboxymethyl)amino]ethyl}(carboxymethyl)amino)acetic acid (EDTA)-treated blood samples were withdrawn from the abdominal vein for flow cytometric analysis. Ten microliters of blood was mixed with 5 μl of phycoerythrin-labeled hamster anti-mouse CD61 antibody (Clone 2C9.G2, BD Biosciences, San Jose, CA, USA) and 15 μl of phosphate-buffered saline in a FACS tube. After 15 min at room temperature, 500 μl of FACS Lysing Solution (BD Biosciences) was added for 30 min to lyse and fix the blood cells. Analyses were performed using a FACSCalibur (BD Biosciences). Data analysis was performed using CellQuest Pro software (ver 6.0, BD Biosciences).

### Hematological and biochemical analyses

Rats were anesthetized using isoflurane, and blood samples were withdrawn from the abdominal vein and abdominal aorta for hematological and biochemical analyses. Hematological parameters were analyzed using a hematology analyzer (Siemens Healthcare Diagnostics Manufacturing Ltd., Deerfield, IL, USA, or Sysmex Corporation, Hyogo, Japan). Blood clotting times were analyzed using an automated coagulation analyzer (Sysmex Corporation, Hyogo, Japan). Blood biochemistry was analyzed using an automated biochemical analyzer (JEOL Ltd., Tokyo, Japan).

### Histological analysis

Bone marrow from the femur and tissues from the spleen, liver, and thymus were excised, fixed with 10% buffered formalin, and embedded in paraffin. Sections were prepared and stained with 4′,6-diamidino-2-phenylindole (DAPI) or hematoxylin and eosin (HE). The DAPI-stained and HE-stained sections were examined using fluorescence and conventional microscopy, respectively.

### Platelet aggregation

Citrated blood was collected from the abdominal aorta and centrifuged to obtain platelet-rich plasma (PRP) (190 × *g*, 10 min at room temperature) and platelet-poor plasma (PPP) (2000 × *g*, 10 min at room temperature). The platelet count in PRP was determined, and the PRP was diluted with PPP to obtain a suspension of 50 × 10^4^/μl platelets. PRP (192 μl) was stirred for 1.5 min at 37°C and 8 μl of ADP (final concentration 20 μM) or collagen (final concentration 10 μg/ml) was added to induce platelet aggregation.

### Intravital imaging

Male CD41-ZsGreen1 transgenic rats were anesthetized by inhalation of 5% isoflurane and then maintained at 2%–2.5% isoflurane. The left saphenous artery was exposed, and a filter paper (1 × 1 mm), presoaked with 10% FeCl_3_ was attached for 3 min to induce thrombosis 2 h after administration of vehicle or prasugrel (3 mg/kg, p.o.). Rats were placed in the prone position and the saphenous artery was attached to a glass plate. The focal plane was then adjusted at the area of widest vessel width where distinct platelet fluorescence images were evident under microscopic observation. Starting 5 min after FeCl_3_ application, fluorescence was measured every 1 s for 25 min using an inverted microscope (TE2000-U, Nikon Corporation, Tokyo, Japan), a spinning-disk confocal laser microscope (CSU-W1; Yokogawa Electric Corporation, Tokyo, Japan), a 20X objective lens, an excitation laser (excitation wavelength 488 nm), and an EM charge-coupled device camera (iXon3 DU888, Andor Technology Ltd., UK) to detect 516 nm fluorescence. The fluorescence image (1024 pixels × 350 pixels) was captured using iQ2 software (Andor Technology Ltd., Belfast, UK), and the 1-min average and the sum of the fluorescence intensity acquired for 25 min were calculated.

### Statistical analysis

Data are expressed as the mean ± standard error of the mean. The statistical analysis of the differences in data between CD41-ZsGreen1 transgenic rats and wild-type SD rats was performed using Student’s *t* test (statistical significance < 5%). Comparisons of intravital imaging of the prasugrel and vehicle groups were performed using Welch’s *t* test (statistical significance < 5%). SAS System Release 9.2 (SAS Institute Inc., Cary, NC, USA) was used to determine statistical significance.

## Supporting Information

S1 Video Intravital imaging video of circulating platelets in saphenous arteries of a CD41-ZsGreen1 transgenic rat. Male CD41-ZsGreen1 transgenic rats were anesthetized, the left saphenous artery was exposed. Fluorescence was measured every 0.05 s using a confocal laser microscope.
(MP4)

S2 Video Intravital imaging video of platelet thrombus formation induced by FeCl_3_ in a vehicle-treated CD41-ZsGreen1 transgenic rat. Male CD41-ZsGreen1 transgenic rats were anesthetized, the left saphenous artery was exposed, and a filter paper, presoaked with 10% FeCl_3_ was attached for 3 min to induce thrombosis 2 h after administration of vehicle. Fluorescence was measured every 1 s for 25 min using a confocal laser microscope. Actual time after FeCl_3_ application is display time + 5 min.
(MP4)

S3 Video Intravital imaging video of platelet thrombus formation induced by FeCl_3_ in a prasugrel-treated CD41-ZsGreen1 transgenic rat. Male CD41-ZsGreen1 transgenic rats were anesthetized, the left saphenous artery was exposed, and a filter paper, presoaked with 10% FeCl_3_ was attached for 3 min to induce thrombosis 2 h after administration of prasugrel. Fluorescence was measured every 1 s for 25 min using a confocal laser microscope. Actual time after FeCl_3_ application is display time + 5 min.
(MP4)
